# Context Dependency in Bark Beetle-Fungus Mutualisms Revisited: Assessing Potential Shifts in Interaction Outcomes Against Varied Genetic, Ecological, and Evolutionary Backgrounds

**DOI:** 10.3389/fmicb.2021.682187

**Published:** 2021-05-12

**Authors:** Diana L. Six, Kier D. Klepzig

**Affiliations:** ^1^Department of Ecosystem and Conservation Science, The University of Montana, Missoula, MT, United States; ^2^The Jones Center at Ichauway, Newton, GA, United States

**Keywords:** context dependency, mutualism, Ophiostomatales, bark beetle, conditionality, by-product mutualism, *Ips*, *Dendroctonus*

## Abstract

Context dependency occurs when biological interactions shift in sign or magnitude depending upon genetic, abiotic, and biotic context. Most models of mutualism address systems where interaction outcomes slide along a mutualism-antagonism continuum as environmental conditions vary altering cost-benefit relationships. However, these models do not apply to the many mutualisms that involve by-product benefits and others that do not have antagonistic alternate states. The ubiquity of such mutualisms indicates a need for different approaches and models to understand how environmental variability influences their strength, stability, and ecological roles. In this paper, we apply the concept of context dependency to mutualisms among bark beetles and fungi that span a variety of life strategies and exposures to environmental variability. Bark beetles and their mutualist fungi co-construct a niche based on by-product benefits that allows them to exist in a resource that is otherwise intractable or inaccessible. For the closest of these partnerships, this has resulted in some of the most influential agents of forest mortality in conifer forests worldwide. Understanding these symbioses is key to understanding their influence on forest structure and dynamics and responses to change. We found no evidence that bark beetle mutualisms change in sign as conditions vary, only in magnitude, and that the “closest” (and most environmentally influential) of these partnerships have evolved behaviors and mechanisms to reduce context-dependency and stabilize benefit delivery. The bark beetle-fungus symbioses most likely to slide along a mutualism-antagonism continuum are those involving loosely associated facultative symbionts that may provide benefits under some circumstances and that are horizontally transmitted by the beetle host. Additionally, some symbiotic fungi are never mutualists – these “third party” fungi are exploiters and may shift from commensalism to antagonism depending on environmental context. Our assessment indicates that a careful differentiation between bark beetle-fungus partnerships is crucial to understanding how they influence forests and respond to environmental variability.

## Introduction

Context dependency, the concept that outcomes of biological interactions shift in sign or magnitude depending upon genetic, abiotic, and biotic context, is core to understanding how interspecific interactions function and how they influence community dynamics and stability. In 2004, we published a paper examining the potential for context dependency in bark beetle-fungus symbioses (Klepzig and Six, 2004). Our review was stimulated by Bronstein’s (1994) paper wherein she posed several key questions for establishing when context dependency occurs. At the time of our paper, massive gaps in knowledge existed in how bark beetle-fungus symbioses functioned and we were only able to touch upon some of these questions. However, in the 17 years that have passed, an increase in interest in these systems, in part driven by massive climate-driven bark beetle outbreaks, has resulted in an explosion of new information. Concurrent with the expansion of literature on bark beetle-fungus symbioses, a growing recognition of the importance of mutualism in shaping biotic communities led to a greater understanding of context dependency in mutualisms in general. Consequently, we felt the time was right to revisit the degree to which context dependency operates in these ecologically and economically important insect-fungus interactions.

There is an increasing recognition that bark beetle (Curculionidae; Scolytinae)-fungus (primarily Ophiostomatales) symbioses vary in specificity and outcome and we now know that these differences play a major role in the extent to which they influence and shape the ecosystems within which they exist (Six, 2020a). Some, like the aggressive tree-killing *Dendroctonus ponderosae*-fungi mutualism (mountain pine beetle) are major drivers of ecosystem structure and function, while non-aggressive secondary beetles and fungi that colonize dying and recently killed trees play less influential roles (Six, 2020a). However, while the beetles are recognized to exhibit a broad range of behaviors, the fungal partners have often been reduced to simple binary categorizations as pathogen/non-pathogen or mutualist/antagonist, and their influence on the host beetle has been studied overwhelmingly in the context of the initial colonization phase of the tree and in tree defense induction. However, the longest period of interaction between a beetle host and its associated fungi occurs over the several months to year-long or longer period following attack. During this time, the fungi express a variety of characteristics that ultimately drive the outcome of the symbiosis. Variation in these characteristics and in the different ecological strategies of the fungi influences the type and strength of the symbiosis, the diversity of partners, and, as we shall discuss, the potential for context dependency.

Our treatment of context dependency in bark beetle-fungus symbioses is split into three sections. The first provides a brief review of what constitutes context dependency, the factors expected to influence it, and the evidence for (or against) it in mutualisms. In the second, we present descriptions of the various types of bark beetle-fungus symbioses and discuss evidence for or against context dependency in their outcomes. In the third, we identify remaining gaps and suggest avenues for further inquiry.

Throughout, we use symbiosis as a general term for organisms living in close association with one another regardless of whether outcomes are positive, neutral, or negative. We use the terms mutualism, commensalism, and antagonism to describe symbiotic outcomes in recognition that some symbioses may shift between these categories and that a strict designation of association type for some can be misleading (Bronstein, 1994). We also limit our treatment to the bark beetle-fungus symbioses that colonize conifers because their associations with fungi are distinct from those of angiosperm-colonizing species and because they are better studied.

## What is Context Dependency in Mutualism?

Context dependency occurs when interaction outcomes shift in magnitude (effect size) and/or sign (+, 0, −) as a function of abiotic, biotic, or genetic context (Chamberlain et al., 2014). We follow here the broadly accepted definition provided by Hoeksma and Bruna (2015) that context dependency is a change in interaction net outcome; in other words, how one species influences the mean fitness correlates of another species. We also follow their reasoning that context dependency should be applied only to interactions between a particular pair of species and exclude changes in the performance of one species when it is paired with an alternate partner species. Thus, if one species pairs with more than one partner, each pairwise interaction should be considered independently. This appropriately restricts comparisons to those occurring within interactions rather than among interactions involving third-party symbionts or hosts although these can, and should be, considered under the umbrella of biotic context (see below).

Context dependency has often been assumed for mutualisms. In fact, mutualisms are routinely viewed as existing on an interaction continuum where outcomes slide between mutualism, commensalism, and antagonism (including parasitism and competition) as conditions (contexts) shift (Bronstein, 1994; Thrall et al., 2007). This view has been reinforced by a large body of research on mycorrhizae and legume-rhizobia and other mutualisms involving nutrient exchange (Hoeksma and Bruna, 2015). However, it is increasingly clear that many mutualisms do not operate on such continua (Lam and Chisholm, 2019). For example, by-product mutualisms, those where benefits accrue as part of the normal function of a partner without additional cost to the provider, and many highly specific obligate mutualisms either do not have alternate states or are highly constrained from shifting to commensalism or antagonism (Bronstein, 1994; Connor, 1995). One of the reasons sign shifts are seldom seen in mutualisms is that when there is a shift to antagonism this is quickly followed by the dissolution of the partnership or lineage extinction (Frederickson, 2017). Furthermore, even in mutualisms that lend themselves to context dependency, shifts in sign may not be as prevalent as was once believed (Karst et al., 2008; Chamberlain and Holland, 2009). A meta-analysis of studies on mycorrhizae found that fungal partners had routinely positive effects on plant growth regardless of shifts in soil nutrient availability (Karst et al., 2008). Another meta-analysis focusing on ant-plant protection mutualisms also found routinely positive effects for plants and only occasional shifts to neutrality (Chamberlain and Holland, 2009). In a broad comparison of mutualisms with plants, Morris et al. (2007) found that the mean effects of pollinator, bacterial, and arbuscular and ectomycorrhizal mutualisms seldom became negative or neutral and generally remained positive.

While shifts in magnitude rather than sign have been more commonly observed, certain types of mutualisms appear to lend themselves more to sign shifts than others. These include plant endophytes and secondary endosymbionts involved in protection mutualisms with insects (Hansen et al., 2007; Oliver et al., 2008). Some endophytic fungi in plants can shift from mutualistic to antagonistic depending on endophyte and/or plant genotype and environmental conditions (Faeth and Fagan, 2002). For example, shifts in soil nitrogen can influence whether endophytes are helpful in defending against herbivores or shift to parasitism (Faeth and Fagan, 2002). Facultative bacterial endosymbionts can increase host plant range, tolerance to heat stress, and provide protection against natural enemies for a number of insects (Oliver et al., 2008; Burke et al., 2010). However, the frequency of some protective secondary endosymbionts is high only when the host insect is under strong pressure but drops drastically when pressure is low suggesting that the symbionts become costly when protection is not needed (Hansen et al., 2007; Oliver et al., 2008).

To determine if context dependency occurs, it is necessary to quantify shifts in the costs and benefits of the interaction on both partners as abiotic, biotic, and genetic contexts vary. Unfortunately, explicit tests have seldom been applied outside of mycorrhizae, *Rhizobium*-legume, ant-plant, and pollinator systems (Hoeksma and Bruna, 2015). Additionally, such cost-benefit analyses are notoriously difficult in many mutualisms. However, particular characteristics of a mutualism provide clues as to whether and when context dependency may be expected and whether a sign change is involved or only changes in the effect size of benefits. Mutualisms are less likely to be context dependent when the environment is predictable, when symbiont richness is low, there is high symbiont reliability, and high quality rewards (Thrall et al., 2007). In particular, predictable and stable environments are thought to lead to increased closeness due to greater predictability of potential partners (Thrall et al., 2007). This, in turn, may lead to selection for increases in benefits over time and a greater potential for obligacy, especially if one or both partners provide access to highly-limiting resources or otherwise alter niche space in a way required for survival of one or both (Buser et al., 2014). On the other hand, an inability to consistently associate with a particular partner, either due to a diverse symbiont pool, high environmental variability, or both, results in a facultative symbiosis and reduces the potential for co-evolution, partner specificity, and dependence (Thrall et al., 2007).

Whether a mutualism is obligate or facultative is a major factor influencing its potential to be context dependent. Obligate mutualisms are constrained from shifting sign because a switch to commensalism or antagonism causes massive reductions in fitness and the extirpation of one or both partners. For obligate mutualisms, context dependency is more likely to be expressed *via* shifts in the effect size of benefits. For example, in a mutualism between a stinkbug and a bacterial symbiont, the symbiont is required for development to adulthood and the symbiosis never switches from positive to neutral or negative because this would result in the death of both partners. However, particular components of the biotic environment such as the species of host plant (potentially through differences in secondary chemistry or nutritional quality) can influence the fitness of the symbiont such that it cascades to affects the growth rate of the host bug (Couret et al., 2019). In contrast, some facultative symbionts may be beneficial in some contexts but neutral or antagonistic in others. These switches in outcome signs do not lead to extinctions because no partner is dependent on the other for survival.

Transmission mode also influences the potential for context dependency. Vertically-transmitted symbionts are the most likely to be specific and obligate and their transmission often involves specialized structures or behaviors that reinforce contact (Six, 2012; Fisher et al., 2016). On the other hand, horizontally-transmitted symbionts are often facultative and with substantial potential for hosts to make new acquisitions or replacements. There is also a tendency for horizontally-transferred symbionts to be antagonistic (pathogens, parasites, and competitors), while those that are vertically transmitted tend toward mutualism because symbiont fitness is dependent on host fitness (Yamamura, 1993; Herre et al., 1999). Seldom addressed in symbiosis theory are mutualisms that involve both modes of transmission or that commonly involve intra-specific and inter-specific host-switching. For example, *Microtermes* termites primarily transmit their fungal symbionts vertically yet frequent horizontal exchange occurs between colonies of the same species and even different species (van de Peppel and Aanen, 2020). Likewise, while some ambrosia beetles exhibit strong partner fidelity with, and vertical transmission of, particular ambrosia fungi, some may acquire fungi directly from their environment and possess different symbionts in different locations (Kostovcik et al., 2015; Rassati et al., 2019). In many cases where both vertical and horizontal transfers occur, there are taxonomic constraints such that symbionts shared among related hosts are also closely-related (Skelton et al., 2019). For instance, host specificity of symbiotic fungi associated with termites in the Macrotemitidae occurs at the genus level but not at the species level (van de Peppel and Aanen, 2020). In such cases, switching may be supported by common needs among hosts that can be provided by a number of related symbionts. Such a “semi-open system” operates as long as mechanisms exist for recognition of “insiders” and avoidance of “outsiders.”

Transmission mode also influences the evolution of costs and benefits to each partner and the potential for context dependency. In general, hosts are predicted to evolve reduced vertical transmission of symbionts that act as antagonists and increased vertical transmission of those that confer benefits (Yamamura, 1993). However, environmental context can play a major role in whether symbiont specificity and vertical transmission develops, even when an interaction results in a strongly mutualistic outcome. For example, mutualisms that operate across large spatial scales may be less likely to become specific and more likely to be context dependent. The geographic mosaic theory of coevolution predicts that fitness consequences of associations should vary across space and time and that selection will result in variable evolutionary outcomes (Thompson, 1994, 2005; Nuismer et al., 2000). This may especially be the case for mutualisms occupying heterogeneous landscapes. In spatially-structured populations that experience different biotic and abiotic contexts, a symbiont may be a mutualist in one environment and neutral or antagonistic in another, such as with the grass *Agrostis hyemalis* where the fungus *Epichloe amarillans* supports greater fecundity in arid locations, but not in areas with wetter conditions, or can decrease grass biomass in the presence of some microbial communities (Davitt et al., 2011; Brown and Akcay, 2018). In some cases, variable environmental conditions may support different symbionts (species or genotypes) in different host populations due to disparities in the needs and growth requirements of hosts, symbionts, or both (Brown and Akcay, 2018). Furthermore, high variability in environmental conditions can result in a variable symbiont pool decreasing the predictability of encountering and developing a specific association with any one symbiont. Such spatially-conditional mutualisms can result in host-symbiont conflict when hosts carrying locally-adaptive symbionts migrate from one population into another (Brown and Akcay, 2018).

Where conditions are stable and shift very little, context dependency in a mutualism is likely to be null or small (Hoeksma and Bruna, 2015). However, when instability reduces the ability of a partner to persist or causes shifts in costs and benefits for one or both partners, context dependency should be more prevalent. Abiotic, biotic, and genetic contexts may all vary to create shifts in symbiotic outcomes. Most symbioses operate under variable abiotic contexts, including, e.g., temperature, moisture, light, climate, and soil nutrient content. Thermal variability, for example, is a common factor influencing interaction strength and outcome. Most organisms are ectotherms and most symbioses involve one or more ectothermic partners. The high sensitivity of ectotherms to temperature can lead to strong and rapid responses in the physiological functions that underlie growth, survival, and the delivery of benefits to mutualist partners (Renoz et al., 2019). Likewise, water is required by all organisms and variability in its form and availability can be another major source of context dependent outcomes (Ruiz-Lozano, 2003). Nutrient acquisition is the driving force behind the formation of most mutualisms and can be influenced by a variety of environmental factors.

In some cases, a mutualism may be largely based on abiotic context such as those that occur between some secondary endosymbionts that confer protection from heat to their insect host (Heyworth et al., 2020). In many other cases, the abiotic context is crucial for aligning partners to function under a common set of conditions. For example, some corals “bleach” when temperatures exceed thresholds for the survival of their dinoflagellate algal symbionts (Cunning et al., 2015). This can be lethal; however, some corals can acquire new heat-tolerant symbionts to replace heat-intolerant ones, allowing the persistence of the symbiosis under new conditions (Cunning et al., 2015). Obligate, co-evolved mutualisms can be expected to possess partners well-aligned to operate under the typical conditions found in their environment, but persistence may be challenged during extreme events or under novel conditions (Six and Bentz, 2007). In contrast, facultative mutualisms are more fluid in their responses to change and may be more open to the acquisition of new partners as conditions shift (Batstone et al., 2018).

Biotic context encompasses all the organisms that the mutualism interacts with directly or indirectly including host plants for herbivores, other symbionts or hosts, predators, parasitoids, and competitors. These are termed third-party effects and they can have substantial consequences for symbiotic outcomes. For example, in ant-plant protection mutualisms with low ant species richness, there is no tendency to be context dependent while those with high ant species richness vary considerably in outcome (Chamberlain and Holland, 2009). With heritable facultative endosymbionts that confer protection to their host insects, coinfection with symbionts that provide different types of defense does not necessarily lead to protection against a broader array of enemies, but rather can result in greater variability in protection or even a reduction such as was seen in pea aphids coinfected by the bacterial symbionts, *Hamitonella* and *Regiella* (Weldon et al., 2020). In contrast, in mycorrhizal systems, the presence of more mycorrhizal fungal species and non-mycorrhizal microbes can increase the nutritional benefit accrued by host plants (Hoeksema et al., 2010). In some cases, predators, parasites, or parasitoids may exploit cues produced by one partner to exploit the other resulting in a decrease in fitness (Adams and Six, 2008; Boone et al., 2008). For example (O’Brien et al., 2018), in mutualisms that exploit plants, variability in the production of secondary chemical compounds within or among plant species can influence benefit delivery by microbial partners (Couret, et al., 2019). The range of third party effects that can occur is immense and they are often very important influences on mutualism outcomes.

Another consideration when assessing context dependency is whether cheaters and exploiters exist in the mutualism. Here, we follow the careful distinctions of Frederickson (2013) between the two. A cheater is a genotype or species that exhibits an adaptive uncooperative strategy, is evolutionarily derived from a cooperator, and reduces partner fitness relative to cooperators. Cheaters are distinct from inefficient partners (those that provide lower fitness benefits due to genotype or defect; Friesen, 2012) and parasites or exploiters of mutualisms which are similar in effect to cheaters but are not evolutionarily derived from cooperators (Frederickson, 2017). Cheaters, by definition, cause a net reduction in fitness. If cheating is influenced by context, this may allow for both a change in sign as well as a change in the magnitude of the interaction outcome. However, evidence for the existence of cheating in mutualisms is sparse (Frederickson, 2017). In contrast, exploiters are commonplace. Exploiters can be especially prone to context dependency and, as with other third parties that exert negative pressure on mutualisms, may be particularly powerful in influencing the dynamics of populations of mutualistic pairs.

Outcomes of symbioses can vary substantially across different genotypes of the symbiont and host. If different genotypes respond to abiotic or biotic contexts in disparate ways, this can result in differential provisioning of benefits. Such genetically based variability provides the potential and foundation for directional selection on mutualism traits (Hoeksma and Bruna, 2015; Stoy et al., 2020). Genotype x environment (GxE) interactions support directional selection in one partner, while genotype by genotype (GxG) interactions of host and symbiont result in co-evolution. Finally, genotype by genotype by environment (GxGxE) interactions support the development of geographic selection mosaics. In microbial symbionts, selection over time is expected to reduce genetic variability by removing all but the best partner genotype(s) (Hoeksma and Bruna, 2015). While this has been observed in many co-evolved mutualisms, it is not always the case (van de Peppel and Aanen, 2020). Genetic variation may be maintained, or its loss slowed, if the optimal mutualist partner remains substantially influenced by the abiotic or biotic environment (Bever, 1999). For example, while endosymbionts may lose large portions of their genomes because the host provides protection and genes to support many crucial functions (McCutcheon and Moran, 2011), ectosymbionts must often contend directly with the external environment and may need to retain full or near full genome function although overall allelic diversity may be reduced (Heath and Tiffin, 2007).

Genetic diversity in symbionts should influence their potential to respond to changing conditions in a context dependent manner. Low genetic diversity may result in greater reliability of benefits and a lower potential for cheating, but it also can constrain a host to particular environmental conditions and habitats because symbionts with low genetic diversity will have narrower ecological amplitudes. On the other hand, symbionts with greater genetic variation may allow for greater flexibility in a variable environment, but increase the potential for context dependent responses by reducing reliability in benefit delivery (Heath and Tiffin, 2007).

Finally, an important consideration is that partners are often subject to many contextual factors simultaneously and these can interact to influence outcomes. Clearly, many factors have the potential to influence outcomes in mutualisms.

## Are Bark Beetle-Fungus Symbioses Context Dependent?

### Background on the Partners

All bark beetles carry fungi during dispersal and develop in their presence within their woody plant hosts. However, not all of these are symbiotic and fewer still are mutualists. Only some bark beetles have consistent or specific fungal partners. Unfortunately, descriptions of fungal symbionts have focused almost completely on tree-killing beetles (primary or aggressive secondary beetles) and little is known about the consistency of fungi with those infesting weak, dying, or newly killed trees (non-aggressive secondary beetles). The effects of highly inconsistent fungi are likely negligible and are not considered here. However, consistent, and especially specific, associations between fungi and beetles can have a marked influence on host beetle fitness. These interactions can best be understood by observing where the partners live and how they use a tree as habitat and food and, for mutualists, the degree and type of niche co-construction (Six, 2020b).

In the initial stages of colonization, beetles and fungi enter a tree that is either living or recently killed and thus, one that is defended. This “defensive period,” wherein the tree challenges the partners with physical barriers and toxic chemicals, can last for days or weeks depending on the state of the tree (weak, vigorous, and freshly-killed) at the time of colonization, species of tree, tree genotype, and the density and rapidity of beetle attacks (Raffa et al., 2015). Beetles first encounter constitutive defenses, such as resin, that pose a formidable physical barrier to entering the tree. Beetles (and their fungi) that get past these initial defenses may then encounter induced responses as the tree upregulates production of secondary chemicals, typically in response to fungal elicitors (Cale et al., 2019). Generally, high numbers of attacks over a short period can circumvent the induced response, while lower numbers of attacks or a drawn-out attack period allows time for the tree to produce localized lesions and resin containing higher levels of toxic secondary chemical compounds that may engulf the invading beetles and contain any further growth by the fungi (Krokene, 2015). However, if the density of beetle attacks is high, the tree will cease resin and defense chemistry production and colonization by the beetles proceeds. Beetles mate and begin to construct galleries within the phloem once defenses decline (or immediately as is the case for non-aggressive secondaries entering dying or recently dead trees). As adults tunnel they lay eggs and inoculate the phloem with fungi. A few days post-oviposition, the eggs hatch and the larvae begin to construct individual galleries (except for a few species that feed communally). Fungal growth is initially slow, but proceeds more rapidly as tunneling within the relatively less defended host tissues increases oxygen and reduces moisture levels. Unlike the beetles, the fungi are not confined to foraging in the phloem and soon begin to grow into the sapwood as well (Six and Elser, 2019).

While beetle-associated fungi share many commonalities, they also have many differences. Even within a taxonomic group, the fungi vary considerably in their ecological strategies. This, in turn, affects their interactions with host trees and beetles. Most fungi considered symbiotic with conifer-colonizing bark beetles are Ascomycota in the Ophiostomatales. These fungi cannot degrade wood and are limited to foraging for sugars, amino acids, and other easily accessed and assimilated compounds. Some are pathogens with varying degrees of virulence (the *relative* ability to cause damage to the tree). Within this large group, three genera are particularly well-represented with bark beetles. *Grosmannia* (*Leptographium* anamorph) species are necrotrophs (pathogens that feed on dead tissues) that range from very weakly to moderately virulent in weakened and apparently healthy trees. *Ophiostoma* species are also necrotrophs but most are only weakly virulent with some bordering on saprotrophy (non-pathogenic, feeding on dead tissues). In contrast, *Ceratocystiopsis* all appear to be saprotrophic. Associations of bark beetles with fungi in these three genera range from incidental to highly specific with no clear relationship between specificity, virulence to the tree, or beetle colonization strategy (aggressive tree killers vs. secondaries that colonize weak, dying, or dead trees). Beetle-associated fungi with high virulence to trees (especially the ability to kill trees on their own) are very rare. The highly virulent fungi are all Microascales in *Endoconidiophora* and their occurrence and incidence is highly variable within and among populations of the spruce beetles *Dendroctonus rufipennis* and *Ips typographus* (Kirisits, 2004; Biedermann et al., 2019). A few bark beetles, mostly in one clade of *Dendroctonus*, carry saprotrophic Basidiomycota in *Entomocorticium*. The basidiomycetes are the only bark beetle-associated fungi capable of degrading cellulose and lignin and some support growth of beetle larvae in outer bark rather than in the typical phloem substrate (Ayres et al., 2000; Six and Elser, 2019; Six, 2020b).

Faced with a diversity of resource acquisition strategies by infecting fungi, trees have evolved corresponding responses. More virulent necrotrophs are able to more rapidly expand into living phloem and then into the sapwood to acquire resources (nutrients and space). They also elicit strong induced defenses from the tree that can negatively affect the survival and fitness of their beetle vectors (reviewed by Hofstetter et al., 2015; Krokene, 2015). As the fungi grow and develop a mycelium, some play a role in detoxifying host tree defenses (Hammerbacher et al., 2013; Wadke et al., 2016; Zhao et al., 2018). This has been found *in vitro* and *in plantae* and this process may alleviate toxic effects on the beetle once the fungi develop sufficient mycelium within the tree (Hammerbacher et al., 2013; Wadke et al., 2016; Zhao et al., 2018). In contrast, weakly virulent necrotrophs and saprotrophs grow more slowly, at least initially, and ultimately elicit smaller total induced responses. For all species, once host defenses are overcome and decline, fungal growth rates increase substantially.

Post-colonization, beetles, and fungi alike must get down to the business of life – the acquisition of sufficient amounts of the proper nutrients to support their growth and reproduction. Both face the challenge of using woody tissues that are highly imbalanced and limiting in many requisite elements, particularly nonstructural carbon (NSC), nitrogen (N), and phosphorus (P). The fungi efficiently focus their growth only on tissues that contain nutrients (phloem and ray parenchyma cells) and cross through the nutritional desert of xylem only rarely and then only to access new rays (Six, 2020b). While sapwood contains low amounts of N, P and NSC, its volume is massive compared to phloem. A fungal mycelium can potentially access the entirety of this resource while a beetle larva is confined to feeding in a small area of phloem (Six and Elser, 2019, 2020). Access to phloem is especially limited in beetle species that pack densely into trees due to mass attack behaviors.

While phloem is higher per unit area in NSC, N, and P than sapwood, it can still be deficient relative to the needs of developing beetles, especially those that colonize trees in high densities. Beetle mutualist fungi mainly colonize and draw nutrients from sapwood and then translocate the N and P absorbed there to the phloem to support reproducyion. This concentration of nutrients into the phloem along with the physical space provided by the pupal chamber supports fungal sporulation in the proximity of emerging vector beetles. This movement of N and P to phloem creates the by-product effect of providing these same nutrients to their beetle host.

Over time, some of these by-product mutualisms have become highly specific, obligate, and highly co-evolved (Bracewell and Six, 2014, 2015; Bracewell et al., 2018). Beetles have invested in the production of mycangia, complex highly selective structures for transporting mutualist fungi (Klepzig and Six, 2004; Bleiker et al., 2009; Bracewell and Six, 2014, 2015). Mycangia appear crucial for vertical transmission and co-evolution between the beetle host and mutualist fungi. These beetles have high N and P demands that can only be met by feeding on the mutualist fungi growing within the phloem substrate (Six and Elser, 2019). Mutualistic fungi, in turn, have evolved to excel at nutrient delivery and invest in the production of dense spore layers in beetle feeding or pupal chambers that are fed upon by newly eclosed adults ensuring their acquisition the mycangia for dissemination, and influencing beetle condition prior to dispersal (Six and Paine, 1998; Six, 2020b). Natural selection also appears to have reduced sexual selection in the fungi reducing variability in benefit delivery (Bracewell et al., 2018).

Non-mutualist fungi are third parties. These fungi typically sporulate more sparsely and often in locations distant to beetles including under bark and in older sections of galleries. These fungi are typically vectored by a variety of arthropods including mites phoretic on bark beetles (Hofstetter et al., 2014). For example, *Ophiostoma minus*, is found with a number of bark beetles world-wide. The fungus is transmitted horizontally, often by phoretic mites and passively on the exoskeleton of beetles. Its incidence in a population can be influenced by beetle population density dependent effects and climatic conditions that regulate vector mite populations (Klepzig et al., 2001). This fungus can compete with the mutualist fungi of some beetles. For example, both *Dendroctonus frontalis* and *Dendroctonus brevicomis* have two mutualist fungi that provide virtually all the food for their larvae. In *D. frontalis* one of these fungi is able to exclude *O. minus* from areas of feeding by beetle larvae (Klepzig and Wilkens, 1997). However, in many cases *O. minus* occurs throughout large areas of phloem and impedes feeding on mutualist fungi and beetle development. In the *D. brevicomis* system, larvae develop in outer bark. *Ophiostoma minus* does not grow into outer bark and larvae developing in its presence die apparently of starvation (Six and Elser, 2019). With some secondary beetles, *O. minus* is not a third party but a mutualist. *Hylurgops porosus* has low N and P demands and can develop in *O. minus*-colonized phloem. Even though this fungus is not highly efficient at concentrating these nutrients, its abilities are sufficient to support the beetle (Six and Elser, 2020).

Like the fungi, bark beetles also exhibit various life strategies, and this influences the type of symbioses they form with fungi (Six, 2020a). We consider these below as we discuss the potential for context dependency in these mutualisms.

### Do Bark Beetle-Fungus Mutualisms Shift in Sign and/or Magnitude?

**Figure 1:**
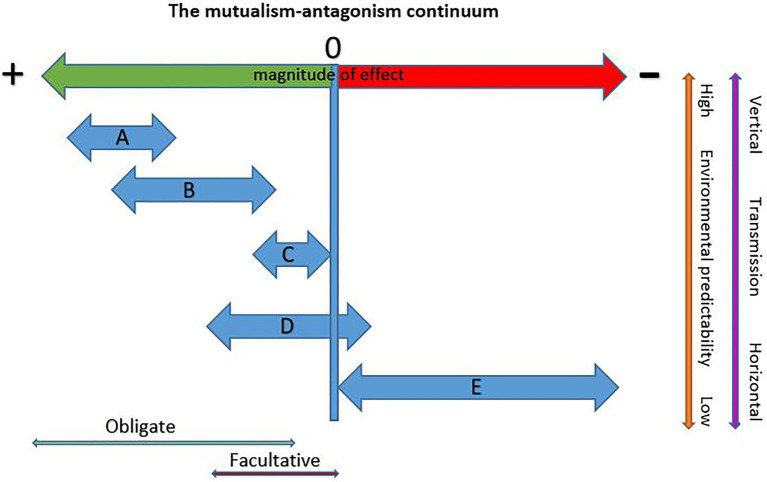
Hypothetical placement of different bark beetle-fungus symbiosis types on the mutualism-antagonism continuum. Potential for context dependency increases with increasing environmental heterogeneity and horizontal transmission. **(A)** Obligate and specific (beetle and fungus) mutualisms with vertical transmission *via* complex structures (mycangia). Typified by aggressive primary beetles requiring high quality nutrient provisioning by fungi and that show a high degree of coevolution with partners. Sign shifts do not occur and magnitude of benefit shifts is highly constrained. **(B)** Obligate (beetle and fungus) mutualisms that may be specific for beetle and fungus or a beneficial fungus may be found with several related hosts. These have mostly vertical transmission *via* simple transport structures (modified pits or setae) and are typified by secondary bark beetles that require high quality nutrient provisioning by fungi. Co-evolution likely. Sign shifts do not occur but the magnitude of benefits may shift contingent with the quality of benefit delivery and availability of phloem. **(C)** Obligate (beetle only), low specificity mutualisms wherein secondary bark beetles exploit fungi brought in by other bark beetles or transport the fungi themselves in simple structures (pits and setae). Co-evolution unlikely and beetles do not require high levels of nutrient provisioning. Sign shifts do not occur but the magnitude of benefits may shift. **(D)** Non-obligate, non-specific interactions involving aggressive or nonaggressive secondary beetles with variable suites of environmentally acquired fungal partners. Because of the unreliable nature of these interactions, the beetle has low-to-no dependence on fungi and primarily gains nutrients from phloem. Shifts in sign possible with some partners. Co-evolution unlikely. **(E)** Third party fungi that may exert little influence or compete with either the beetle and/or its mutualist fungi or otherwise negatively affect fitness. Sign shifts, if they occur are from commensal to antagonist and the magnitude of effects may shift from negligible to highly antagonistic depending on the third party.

To answer this question we need to consider differences among the various bark beetle symbioses with fungi and the degree to which they are exposed to, and can weather, the destabilizing effects of context dependency. Figure 1 presents five types of bark beetle-fungus interactions (A–E) and their hypothesized placement on the mutualism-antagonism continuum. Under this conceptual framework, bark beetles in obligate mutualisms are the most likely to be co-evolved with their fungi and these partnerships cannot shift in sign, but can shift in magnitude of benefits within bounds (Figures 1A,B). Type A beetle-fungus mutualisms occur with some aggressive primary tree-killing beetles and are characterized by high specificity, low symbiont pools, coevolution, vertical transmission, and high quality rewards. Type A beetles have high colonization densities due to mass attack behaviors that reduces phloem availability. These consume low amounts of phloem resulting in short larval feeding galleries. This is possible because the N and P demands of these beetles are met by their fungi (Ayres et al., 2000; Bleiker and Six, 2007; Six and Elser, 2019), Thus, shifts in nutrient quality (reward size) are highly constrained (Figure 1A) as any reduction in provisioning creates immediate strong negative feedbacks on the fitness of both partners. These mutualisms have several characteristics that increase habitat predictability reducing conditionality. Type A beetles typically specialize on one or a few related tree species reducing variability in quality and quantity of tree defenses and nutrients. Using live trees (even if only initially live) reduces the symbiont pool to only those capable of contending with tree defenses, high moisture, and low oxygen conditions present at the time of attack. Early resource capture by these fungi reduces exposure to a variable symbiont pool given they are moving into a “blank slate” reducing the potential for replacements or invasions. Low sexual recombination in high-performing mutualists (Bracewell et al., 2018) increases predictability in reward delivery and the presence of highly selective mycangia in host beetles (Bleiker et al., 2009; Bracewell and Six, 2015) ensures vertical transmission reinforcing stability.

The obligacy of the partners may also feedback to reduce environmental heterogeneity. In a one beetle-one mutualist system, the partner with the most restricted environmental tolerances (typically the fungus) will determine the mutualisms’ overall niche space resulting in a more predictable, but narrower realized niche. However, ecological amplitude increases when a host partners with additional mutualist fungi, each with different tolerances. For example, two mutualists of *D. ponderosae* (*Grosmannia clavigera* and *Leptographium longiclavatum*) grow best when conditions are intermediate to cool while a third (*Ophiostoma montium*) grows best under intermediate and warmer conditions. This results in different distributions and prevalences of each fungus over the beetle’s range and within populations over seasons and years. While the warm-tolerant species provides lower quality rewards to the host, it is entirely capable of supporting beetle growth and development and its presence allows the beetle (and the mutualism as a whole) to contend with more environmental variability although within bounds. When conditions are too hot, even the warm-tolerant fungus of *D. ponderosae* does not sporulate and brood adults disperse without maturation feeding on spores and without fungi for their young (Bleiker and Six, 2008). Thus, while the beetle can tolerate hotter conditions than can its fungi, its realized ecological amplitude is restricted to that of its fungi.

Type B mutualisms occur with aggressive secondary beetles that colonize weak or dying trees. These beetles have generally been assumed to transmit fungi horizontally and to have little-to-no dependence on them and co-evolution is not predicted. However, recent studies reveal a more complex picture. Some of these beetles not only consistently carry fungal associates (suggesting vertical transmission) but also appear to be dependent on their fungi for nutrient provisioning. For example, *Ips pini* and *Ips emarginatus* each carry a consistent fungus, and both have very high demands for N and P that cannot be met feeding on phloem alone (Six and Elser, 2020). However, these and other Type B beetles appear to consume more phloem than Type A beetles (visual comparisons of galleries of North American bark beetles; Wood, 1982) which may allow for greater shifts in magnitude of reward quality through increased phloem consumption (Figure 1B). For example, another B type beetle, *Dendroctonus pseudotsugae*, carries two fungi that likely contribute to its nutrition but also feeds extensively in phloem. Many of these beetles use aggregation pheromones and can colonize trees in high densities, suggesting that they may require relatively high quality rewards from their fungi when competition for phloem is high.

Like Type A beetles, Type B beetles are typically specialized on a particular tree species or a few related trees in one genus reducing environmental heterogeneity. For example, *D. pseudotsugae* (two fungi) is a specialist on *Pseudotsuga menziesii*, *D. rufipennis* (one fungus) is a specialist on *Picea* (Six and Bentz, 2003), and *Ips confusus* (one fungus; Six et al., unpublished data) specializes on *Pinus edulis* and other pinyon pines (Wood, 1982). Entry into living or freshly killed trees reduces exposure to non-mutualist fungi although defenses are often reduced due to stress on, or recent mortality of, the tree. The fungi found with these beetles are typically outcrossing and thus are more genetically diverse and thus variable in reward delivery. Most of the beetles of this type lack complex mycangia, although some possess complex pits or setae that may be considered mycangia. For example, *Scolytus ventralis* has complex glandular pit mycangia that transport its mutualist fungus, *Trichosporium symbioticum* (Livingston and Berryman, 1972). This fungus is consistently associated with the beetle across its entire range suggesting an ancient and co-evolved mutualism (Six et al., unpublished data).

Type C mutualisms involve non-aggressive secondary beetles. Since these are almost completely unstudied in the context of fungal associates, we can only speculate on their structure and outcomes. These beetles colonize recently dead trees, often killed by other bark beetles, disease, or disturbance such as wind or fire. They enter a tree after defenses have declined and some “fill in” gaps between the galleries of aggressive bark beetles and often several species will colonize the tree together. This late entry into a tree exposes them to a larger symbiont pool (due to the combined pool of fungi carried by multiple beetle species as well as early invading saprophytes) and to competition from other beetles and fungi for phloem resources. Low levels of tree defenses allow them to be more generalized and many colonize several species of trees, often from more than one genera. These weak and dying trees are also often simultaneously colonized by several secondary beetle species and their fungi, increasing exposure to a larger potential symbiont pool, and potentially decreasing the ability of any one beetle to specialize on any one fungus. Few Type C beetles have been investigated for a role of fungi in nutrient provisioning. One, *H. porosus*, colonizes *Pinus* and *Picea* (Wood, 1982) often co-occurring with aggressive beetles. The beetle contains very low levels of N and P indicating a low demand for these elements from its diet. In trees colonized by *D. brevicomis*, it commonly feeds in areas colonized by *O. minus*, a fungus that translocates N and P with lower efficiency than the mutualist fungi of aggressive beetles (Six and Elser, 2020). Type C beetles likely hover near the neutral portion of the mutualism-antagonism continuum, requiring only low quality rewards to survive. Low N and P demand coupled with a “scavenger” strategy may require these beetles to be a jack-of-all-trades that can exploit a variety of fungi including low reward partners.

Beetles that have low-to-no dependence on fungi and associate with variable suites are the most likely to experience shifts in sign and magnitude (type D mutualisms; Figure 1D). These beetles typically consume large amounts of phloem reducing dependence on fungi for diet supplementation and moderating the effects of partner variability. Since these symbioses are uninvestigated in the context of nutrient provisioning, we can only speculate as to their context dependency. Any positive effects that occur are facultative given that the beetles and fungi can survive independent of one another. Co-evolution to reduce negative effects and enhance benefits is not supported because of a lack of consistent association and high levels of horizontal transmission. It is possible that some of the fungi impart nutritional benefits to the beetle but whether sign shifts occur is unknown. One example of a Type D beetle is *Ips typographus*, an aggressive tree-killing secondary that carries a highly variable suite of fungi within and among populations across its range (Kirisits, 2004; Biedermann et al., 2019). The fungi are carried in pits but these pits carry several species of fungi and are not apparently selective (Furniss et al., 1990) and thus should not be considered mycangia (although further investigations of these pits may be warranted). Some of the fungi may depend on the beetle for dissemination such as *Endoconidiophora polonica*, a fungus that is variable in its presence with the beetle, but that has not been found outside of the symbiosis. Most others associated with this beetle are carried by a number of other bark beetle species including some beetles that co-occur with *I. typographus*. Larval galleries are long and the larvae feed on large amounts of phloem before pupation. Which fungus or fungi a given beetle develops with is likely a matter of chance combined with differential climatic effects on fungal growth and sporulation. Given that natural selection occurs at the individual beetle level, this indicates that *I. typographus* individuals experience a mosaic of fungal effects depending on which fungus (or fungi) they happen to develop with. However, type D beetles are among the least explored in regard to nutritional mutualisms with fungi. Research investigating how “suites” of fungi can affect a beetle’s fitness and more attention to pits as potential “mycangia” are needed to categorize these species more confidently.

Finally, third-party fungi (type E; Figure 1E) are horizontally transmitted by the host beetle or are introduced into the system by mites or other vectors and do not provide benefits to the beetle. These are not associated with mycangia and their effects may range from null to strongly negative. The magnitude of effects may vary considerably for some, particularly those that compete with the beetle or mutualist fungi for nutrients or otherwise influence the fitness of the beetle and its fungi. For example, if temperature shifts influence the growth of a competitor of a beetle’s mutualist fungi, the interaction may become more or less negative depending on the direction of change. Beetles may have little ability to escape third party effects if they are driven by factors independent of the mutualism.

For bark beetle mutualisms, we have little insight into multigenerational effects of fungal feeding or lack thereof. For Type A beetles, the effect is clear – a lack of fungi during development is lethal. But for some, the lack of an appropriate fungus may only become apparent over multiple generations. For example, some can develop without fungi but produce more brood when fed their mutualist partners. This is the case for *Hylastes* that feed on *Leptographium* and *Ips avulsus* that feeds on its full fungal complement (Yearian et al., 1972). However, while these beetles are capable of development without fungi, lower fecundity could have serious implications for a population over time. What is clear is that different bark beetles have different dependencies on fungi for N and P and that fungal species vary greatly in their efficiency in provisioning these elements.

## Conclusion and Future Directions

The consequences of context dependency are not trivial as some mutualisms are powerful drivers of ecosystem structure and function and conditionality of interaction outcomes may have effects that extend far beyond direct effects on partners. While some mutualists are always mutualists and some parasites are always parasites, some symbionts, particularly facultative ones, may fall on a sliding scale. However, there is currently no evidence that bark beetle mutualisms change in sign, only in magnitude, and that the “closest” of these partnerships have evolved mechanisms to reduce context-dependency and stabilize benefit delivery. The bark beetle-fungus symbioses most likely to slide along the mutualism-agonism continuum are those involving facultative symbionts that occur in suites. However, we know little about these and they should be a priority in future work. Our understanding of bark beetle-fungus symbioses will also be improved by studying the “full” complement of fungi found in mutualisms with beetles. Many mutualisms are multipartite and involve two or more mutualist fungi. However, preconceived notions as to their function or value to the host have often led to a biased selection of one fungus over others, even in obligate mutualisms, leaving a gaping hole in our understanding of the mutualism as a whole. There has especially been the case with Type A beetles where studies are often biased toward the most virulent partner or the one best at provisioning nutrients. However, in obligate mutualisms, all partners are co-evolved with the host and are integral to the full function of the partnership.

One reason we lack a better understanding of many of these mutualisms is that they are notoriously difficult to manipulate in controlled experiments. However, the use of molecular community analyses and ecological stoichiometric approaches can help unravel the roles of fungi in providing nutrients and the use of isotopes can further aid in our understanding of where elements that end up in beetles originate. Knowledge of element source and demand can be used in modeling to predict how context dependency influences outcomes and to detect the point at which it destabilizes the system.

While we have come a long way in understanding these systems since our 2004 paper, we still have a way to go. We are also at a time when understanding the nature of *both* the context *and* the dependency in these mutualisms is needed more than ever so that we can understand how they will respond to anthropogenic change. Bark beetles and their mutualist fungi co-construct a niche that allows them to exist in a resource that is otherwise intractable or inaccessible. For the closest of these partnerships, this has resulted in some of the most influential agents of forest mortality in conifer forests worldwide (Six, 2020a). These fully ectothermic partnerships are influenced by climate at all levels indicating that shifting climatic context will have a major impact on their function and stability of their dependency, and how they influence forests into the future.

## Data Availability Statement

The original contributions presented in the study are included in the article/supplementary material, further inquiries can be directed to the corresponding author.

## Author Contributions

DS developed the overall conceptual framework and wrote the initial draft. KK provided the feedback and additional development of conceptual arguments and co-wrote the final draft with DS. All authors contributed to the article and approved the submitted version.

### Conflict of Interest

The authors declare that the research was conducted in the absence of any commercial or financial relationships that could be construed as a potential conflict of interest.
